# Inhibition of TRIM32 by ibr‐7 treatment sensitizes pancreatic cancer cells to gemcitabine via mTOR/p70S6K pathway

**DOI:** 10.1111/jcmm.17109

**Published:** 2021-12-17

**Authors:** Bo Zhang, You‐you Yan, Yang‐qin Gu, Fei Teng, Xu Lin, Xing‐lu Zhou, Jin‐xin Che, Xiao‐wu Dong, Li‐xin Zhou, Neng‐ming Lin

**Affiliations:** ^1^ College of Pharmaceutical Sciences Hangzhou First People's Hospital Zhejiang Chinese Medical University Hangzhou China; ^2^ Department of Clinical Pharmacology Key Laboratory of Clinical Cancer Pharmacology and Toxicology Research of Zhejiang Province Affiliated Hangzhou First People's Hospital Cancer Center Zhejiang University School of Medicine Hangzhou Zhejiang China; ^3^ Department of Thoracic Surgery School of Medicine The First Affiliated Hospital Zhejiang University Hangzhou Zhejiang China; ^4^ Hangzhou Hezheng Pharmaceutical Co. Ltd Hangzhou Zhejiang China; ^5^ Hangzhou Institute of Innovative Medicine College of Pharmaceutical Sciences Zhejiang University Hangzhou China; ^6^ Innovation Institute for Artificial Intelligence in Medicine Zhejiang University Hangzhou China; ^7^ Department of Hepatopancreatobiliary Surgery Affiliated Hangzhou First People's Hospital Zhejiang University School of Medicine Hangzhou China

**Keywords:** Gemcitabine, Ibr‐7, mTOR, Pancreatic cancer, TRIM32

## Abstract

Pancreatic cancer is one of the most notorious diseases for being asymptomatic at early stage and high mortality rate thereafter. However, either chemotherapy or targeted therapy has rarely achieved success in recent clinical trials for pancreatic cancer. Novel therapeutic regimens or agents are urgently in need. Ibr‐7 is a novel derivative of ibrutinib, displaying superior antitumour activity in pancreatic cancer cells than ibrutinib. In vitro studies showed that ibr‐7 greatly inhibited the proliferation of BxPC‐3, SW1990, CFPAC‐1 and AsPC‐1 cells via the induction of mitochondrial‐mediated apoptosis and substantial suppression of mTOR/p70S6K pathway. Moreover, ibr‐7 was able to sensitize pancreatic cancer cells to gemcitabine through the efficient repression of TRIM32, which was positively correlated with the proliferation and invasiveness of pancreatic cancer cells. Additionally, knockdown of TRIM32 diminished mTOR/p70S6K activity in pancreatic cancer cells, indicating a positive feedback loop between TRIM32 and mTOR/p70S6K pathway. To conclude, this work preliminarily explored the role of TRIM32 in the malignant properties of pancreatic cancer cells and evaluated the possibility of targeting TRIM32 to enhance effectiveness of gemcitabine, thereby providing a novel therapeutic target for pancreatic cancer.

## 1 INTRODUCTION

Pancreatic ductal adenocarcinoma (PDAC) is one of the most aggressive cancer, and is also notorious for its high malignancy and mortality after diagnosis.^1^, ^2^ Since most PDAC patients are diagnosed at late stage, systemic chemotherapy and targeted therapy are considered as important intervention strategies for PDAC patients.^3^, ^4^ Although tremendous efforts have been devoted to explore efficacious compounds for treating PDAC, there are still few regimens that have achieved desired outcomes in clinical studies.^5^ It is assumed that the hyperactivation of both MEK/ERK and AKT/mTOR pathways in approximately 90% of PDAC patients accounts for their insensitivity to cancer drugs.^6^, ^7^, ^8^ Inhibition of either pathway would be insufficient to restrain the proliferation of PDAC cells, except for those possess unique mutant aberrations that could be targeted by specific agents.^9^, ^10^ On the contrary, although gemcitabine remains as the first‐line chemotherapy in PDAC treatment for decades, strategies to improve the efficacy of gemcitabine are facing numerous obstacles and the results are still challenging.^11^, ^12^, ^13^ Therefore, to discover novel agents that could efficiently inhibit the proliferation of PDAC or investigate key components that play decisive roles in gemcitabine sensitivity is extremely compelling in PDAC therapy.

Ibrutinib is an irreversible inhibitor of Bruton's tyrosine kinase, thus stalling the development and maturation of B cells.^14^, ^15^ In pancreatic adenocarcinoma, ibrutinib was found to exert potent antitumour activities in xenograft models.^16^ Unfortunately, ibrutinib failed to corroborate the efficacy of nab‐paclitaxel plus gemcitabine in RESOLVE trial (PCYC‐1137) last year, which put the applications of ibrutinib for PDAC into a dilemma. In our previously published work, where the structure of ibr‐7 was referred, we have described ibr‐7 as a novel derivative of ibrutinib. Ibr‐7 exhibited its superior antitumour activity than ibrutinib via sufficient suppression of EGFR signalling pathway in non‐small cell lung cancer cells.^17^ In our recent work, we have found that ibr‐7 was capable to increase the radiation‐induced cell death by enhancing substantial DNA damage in PANC‐1 and Capan2 cells.^18^ However, the underlying mechanisms of ibr‐7 in PDAC cells, and more importantly, whether ibr‐7 could enhance the effectiveness of gemcitabine remain unclear. In another aspect, although previous reports have demonstrated the involvement of TRIM32 in progression of many types of malignant tumours,^19^, ^20^ its role in PDAC is yet fully investigated. In this study, we attend to fully explore the inhibitory effects and mechanisms of ibr‐7 and meanwhile validate the potential role of targeting TRIM32 to enhance the efficacy of gemcitabine in PDAC cells.

## 2 MATERIALS AND METHODS

### 2.1 Reagents

RPMI‐1640 medium and foetal bovine serum (FBS) were purchased from Gibco (Grand Island, NY, USA). DAPI was purchased from Wuhan Goodbio Technology Co., Ltd (Wuhan, China). The Annexin V‐FITC Apoptosis Kit was purchased from BestBio (Shanghai, China). The Mitochondrial Membrane Potential Assay Kit was purchased from Signalway Antibody (College Park, MD, USA). The primary antibodies against poly(ADP‐ribose) polymerase (PARP), procaspase‐9, procaspase‐3, cleaved caspase‐3, PI3K‐p110ɑ, p‐mTOR (S2448), mTOR, p‐p70S6, p70S6, p‐S6 (240/244), p‐S6 (235/236), S6, p‐ERK and ERK were purchased from Cell Signaling Technology (Beverly, MA, USA). The primary antibodies against X‐linked inhibitor of apoptosis protein (XIAP), Bax, Noxa, Bcl‐XL, Bcl‐2, Mcl‐1 and β‐actin were purchased from Abcam Inc. (Cambridge, MA, USA).

### 2.2 Cell culture

Human pancreatic cancer cell lines BxPC‐3 (Catalog No. TCHu12), SW1990 (Catalog No. TCHu201), CFPAC‐1 (Catalog No. TCHu112), AsPC‐1 (Catalog No. TCHu8) and PANC‐1 (Catalog No. TCHu535) were purchased from the Chinese Academy of Sciences (Shanghai, China). Cells were cultured in RPMI‐1640 or Dulbecco's Modified Eagle Medium (DMEM) containing 10% foetal bovine serum and 100 U ml^‒1^ penicillin/streptomycin at 37°C in 5% CO_2_ in a humidified atmosphere. Ibr‐7, ibrutinib and gemcitabine were dissolved in DMSO at a concentration of 10 mM respectively.

### 2.3 Cell Viability assay

Cell viability was analysed by Cell Counting Kit‐8 (CCK‐8) assay (Bestbio, Shanghai, China). Cells were cultured in 96‐well plates at a density of 6 × 10^3^/well for 24 h. Then, cells were treated with indicated concentrations of compounds for 48 h. Supernatant was totally removed, and 100 μl of CCK‐8 solution was added to each well and cultured for another 2 h at 37℃. Cell viability was quantified by a SpectraMax M2e (Molecular Devices, San Jose, CA, USA) at 450 nm. Cell viability was calculated for each well as (OD450 treated cells/OD450 control cells) ×100%. Assays were performed on three independent experiments.

### 2.4 Apoptosis assay

Cells were seeded in 6‐well plates (2 × 10^5^/well) and cultured overnight in a 5% CO_2_ atmosphere at 37℃. After treatment with ibrutinib, ibr‐7, gemcitabine or the combination for 24 h, cells were harvested and washed with PBS. Then, cells were stained with Annexin V‐FITC Apoptosis Kit according to the manufacturer's instructions and analysed by flow cytometry (Becton Dickinson, Franklin Lakes, NJ, US). Assays were performed on three independent experiments.

### 2.5 Western blot Analysis

After treated with different concentrations of compounds, total proteins were extracted using RIPA lysis buffer. A total amount of 40 μg proteins were subjected to 12% SDS‐PAGE and transferred to PVDF membrane (Bio‐Rad, Hercules, CA, USA). The membranes were blocked with 5% non‐fat milk at room temperature for 1 h and then incubated with primary antibodies overnight at 4℃. After washing with Tris‐buffered saline with Tween 20, membranes were incubated with secondary antibodies at room temperature for another 1 h. The protein bands were visualized by adding ECL system WBKLS0050 (EMD Millipore, Billerica, MA, USA) and analysed using Bio‐Rad Laboratories Quantity One software (Bio‐Rad, Hercules, CA, USA).

### 2.6 DAPI stain

BxPC‐3 and SW1990 cells (8 × 10^4^ cells/well) were cultured in 24‐well plates. After exposure to ibr‐7, gemcitabine or the combination, cells were fixed with 4% paraformaldehyde for 20 min and stained with DAPI for 15 min. After washing with PBS, cells were observed under a fluorescence microscope (Nikon, Ti‐E, Japan).

### 2.7 Detection of the mitochondrial membrane potential

The mitochondrial membrane potential was visualized by 5,5’,6,6'‐tetrachloro‐1,1’,3,3’ tetraethyl‐imidacarbocyanine iodide (JC‐1) staining. BxPC‐3 and SW1990 cells were seeded into 6‐well plates at a density of 2 × 10^5^/well and cultured for 24 h. After 12 h of treatment, the cells were collected, washed with PBS and incubated with JC‐1 for 15 min at 37℃. After washing off the dye, the cells were immediately analysed using flow cytometry (Becton Dickinson, Franklin Lakes, NJ, USA). Assays were performed in three independent experiments.

### 2.8 mRNA library construction and sequencing

Total RNA was extracted using TRIzol reagent (Invitrogen, USA) and purified by poly‐T oligo–attached magnetic beads. mRNA was then fragmented into small pieces using divalent cations under elevated temperature. Then, the cleaved RNA fragments were reverse‐transcribed to create the final complementary DNA (cDNA) library in accordance with the protocol for TruSeq RNA Sample Preparation v2 (catalogs RS‐122–2001 and RS‐122–2002, Illumina, USA). The average insert size for the paired‐end libraries was 300 ± 50 base pairs (bp). Then, we performed the paired‐end sequencing on an Illumina X10 (San Diego, CA, USA) at the LC Biotechnology Co. (Hangzhou, Zhejiang, China).

### 2.9 Small interfering RNA knockdown and transfection

Small interfering RNA (siRNA) targeting TRIM32 and scrambled siRNA were purchased from Guannan Biotechnology (Hangzhou, China). Cells were seeded in 6‐well plates (2 × 10^5^ cells/well). Cells were then transfected with the siRNA using jetPRIME (Polyplus, NY, USA) according to the manufacturer's instructions. The sense sequences of the TRIM32 and control siRNA were 5′‐UGAAGUUGAGAAGUCCAAUAGTT‐3′ (TRIM32 siRNA‐1), 5′‐AUAACUCCCUCAAGGUAUAUATT‐3′ (TRIM32 siRNA‐2); 5′‐GCCACUUCUUCUCGGAGAAUGTT‐3′ (TRIM32 siRNA‐3); 5′‐UUCUCCGAACGUGUCACGUTT‐3′ (Scrambled siRNA).

### 2.10 Virus production and transfection

The 293T cells were seeded into 100 mm × 20 mm dishes. Until they reached 70 ~ 80% confluence, the plasmids encoding recombinant TRIM32 genomes were co‐transfected with expression plasmids psPAX2 and pMD2G into the 293 T cells using PEI (Polysciences, Warrington, PA USA). After 18 h, 10 ml of the virus induction medium was added into the 293T cells to replace the old medium. After 24 h incubation, the supernatants were clarified by centrifugation and stored in aliquots at −80°C. Then, BxPC‐3 or SW1990 cells were transfected with the viruses using hexadimethrine bromide (Sigma, USA).

### 2.11 Real‐time reverse transcription‐quantitative polymerase chain reaction (RT‐qPCR)

Total RNA was extracted from cells with TRIzol, precipitated with isopropyl alcohol and rinsed with 70% ethanol. Single‐strand cDNA was prepared from the purified RNA using oligo (dT) priming (Invitrogen, Thermofisher scientific, Waltham, MA, US), followed by SYBR‐Green (Qiagen, Hilden, Germany) and carried out using 7900HT Fast Real‐Time PCR system (Applied Biosystems Inc., CA, USA). Assays were performed on three independent experiments. The primers used are as follows:

TRIM32, forward primer: 5’‐AGGGGATACACAAGCCCTTT‐3’,

reverse primer: 5’‐TCTCAATCCAAGATGGCACA‐3’;

GAPDH, forward primer: 5’‐GAGTCAACGGATTTGGTCGT‐3’,

reverse primer: 5’‐TTGATTTTGGAGGGATCTCG‐3’.

### 2.12 Transwell migration assay

Cell migration assays were performed using 24‐well Transwells (8 μm pore size, Corning, USA) uncoated with Matrigel. In total, 1  ×  10^5^ cells were suspended in 500 μl RPMI 1640 containing 1% FBS and added to the upper chamber, while 750 μl RPMI 1640 containing 10% FBS was placed in the lower chamber. After 48 h of incubation, cells remaining in the upper chamber were removed using cotton swabs. Cells on the lower surface of the membrane were fixed in 4% paraformaldehyde and stained with 0.5% crystal violet. Cells in 5 microscopic fields (at ×200 magnification) were counted and photographed. All experiments were performed in triplicate.

### 2.13 Colony formation assay

BxPC‐3 cells were seeded into 6‐well plates at a density of 1x10^3^ /well and incubated for 24 h. The cells were then incubated with siTRIM32 or siControl. Following 24 h of treatment, the supernatant was removed and cells were cultured for a further two weeks. Then, the cells were fixed with 4% paraformaldehyde for 15 min and stained with Giemsa solution for 15 min at room temperature. Visible colonies were imaged with a ChemiDoc XPS system (Bio‐Rad Laboratories, Inc., Hercules, CA, USA).

### 2.14 Tumour xenografts assay

All animal experiments were conducted according to the Institutional Animal Care and Use Committee (IACUC). Total amount of 5 × 10^6^ BxPC‐3 cells were resuspended in 200 μl PBS and injected subcutaneously into each 4‐week‐old female nude mice. Once the tumour volume had reached 50 mm^3^, six mice were randomized into each group. Both gemcitabine and ibr‐7 were dissolved in 0.125 ml of DMSO and vortexed for 10 min. Then, 2.375 ml of 20% HP‐beta‐cyclodextrin was added into the above mixture to make a final concentration of 10 mg ml^‒1^. Gemcitabine or Ibr‐7 were administrated intraperitoneally every two or three days at the dose of 30 or 60 mg kg^‒1^ respectively. Tumour volumes were determined from calliper measurements of tumour length (L) and width (W) according to the formula (L × W^2^)/2.

### 2.15 Statistical analysis

The results are expressed as the mean ± SD of at least three independent experiments. Differences between means were analysed using Student's t test and were considered statistically significant when *p *< 0.05. Statistical analyses and data visualization were performed using IBM SPSS version 22.0 (IBM SPSS, Inc., Chicago, IL, USA) and GraphPad Prism, Version 6.01 (GraphPad Software Inc., San Diego, CA, USA).

## 3 RESULTS

### 3.1 Ibr‐7 inhibited the proliferation of PDAC cells via suppression of mTOR and ERK phosphorylation

**FIGURE 1:**
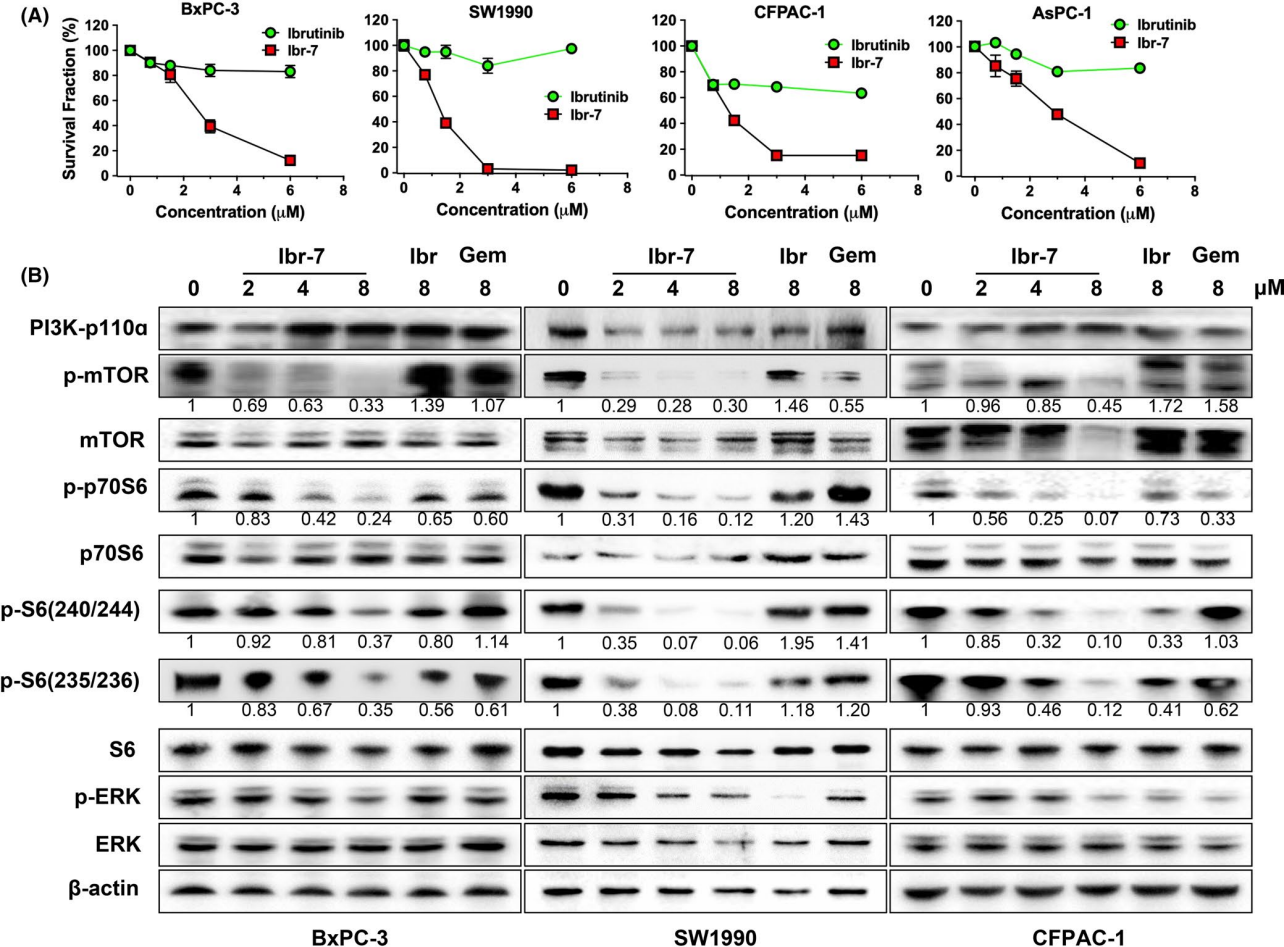
Anti‐proliferative activity of ibr‐7 and its inhibitory effects on mTOR/p70S6K pathway in pancreatic cancer cells. (A) The dose‐dependent inhibitory effect of ibrutinib and ibr‐7 on five pancreatic cancer cell lines in vitro. Cells were treated with ibrutinib or ibr‐7 for 48 h before CCK‐8 assay. (B) Ibr‐7 suppressed phosphorylated proteins in mTOR/p70S6K pathway. BxPC‐3, SW1990 and CFPAC‐1 cells were treated with indicated concentrations of compounds for 8 h before Western blotting analysis. Three independent experiments were performed, and data were presented as mean ± SD

The anti‐proliferation effects of ibr‐7 or ibrutinib were determined in PDAC cells by using CCK‐8 assays. In four tested PDAC cell lines, ibr‐7 showed stronger anti‐proliferation activity than ibrutinib (Figure 1A), and there was a 10 fold to 60‐fold difference between ibr‐7 and ibrutinib in IC50 values (Table 1). Aiming to explore the underlying mechanisms of ibr‐7, we examined essential proteins involved in regulating proliferation in three pancreatic cancer cell lines. As a result, 8 μM of ibr‐7 effectively suppressed phosphorylated mTOR, p70S6 and S6, which were slightly influenced by ibrutinib or gemcitabine treatment (Figure 1B). Besides, phosphorylated ERK was remarkably suppressed after treatment with 8 μM of ibr‐7 or ibrutinib in BxPC‐3, SW1990 and CFPAC‐1 cells. Therefore, the dual‐inhibitory effect of ibr‐7 on both mTOR/p70S6K and ERK might contribute to its potent anti‐proliferation activity in PDAC cells.

**TABLE 1:** The IC50 values of ibrutinib and ibr‐7 on four pancreatic cancer BxPC‐3, SW1990, CFPAC‐1 and AsPC‐1 cells

Cell line	IC50 (µM)	
	Ibrutinib	Ibr−7
BxPC−3	130.3 ± 8.9	3.5 ± 0.2
SW1990	60.7 ± 11.1	0.9 ± 0.1
CFPAC−1	46.1 ± 2.1	1.0 ± 0.1
AsPC−1	24.0 ± 0.9	2.1 ± 0.4

### 3.2 Ibr‐7 induced mitochondrion‐mediated apoptosis in PDAC cells

**FIGURE 2:**
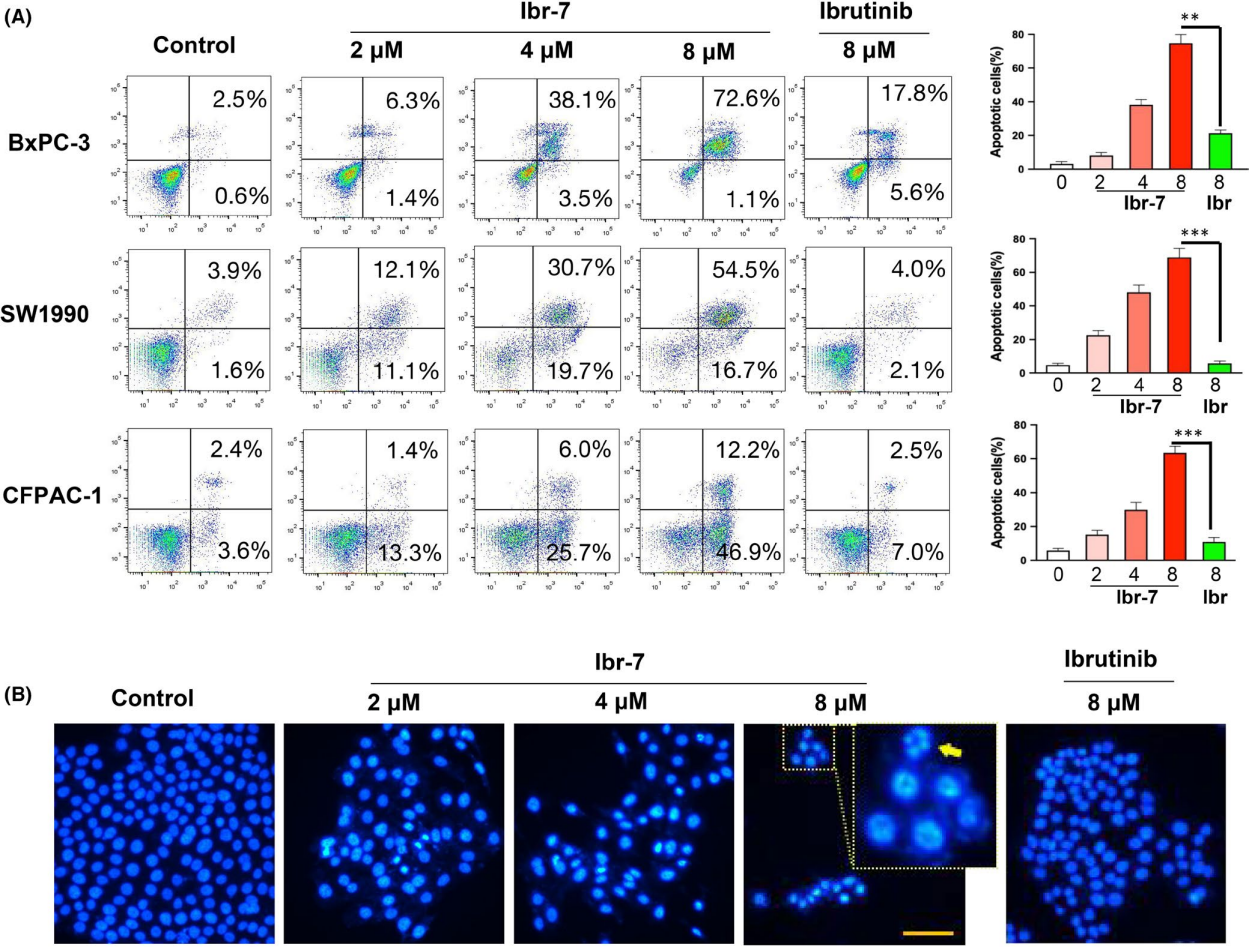
Ibr‐7 induced apoptosis in BxPC‐3 and SW1990 cells. (A) Cells were treated with ibr‐7 or ibrutinib for 24 h before collection. Then, cells were stained by Annexin V/PI and analysed by flow cytometry. (B) BxPC‐3 cells were treated with 2, 4 and 8 µM of ibr‐7 and 8 µM of ibrutinib for 24 h before fixation and stained with DAPI stain. Fluorescence was observed by using microscopy (Nikon Eclipse Ti). The magnification is ×400. Scale bar =20 µm. Three independent experiments were performed, and data were presented as mean ± SD. Student's t test was used to make a comparison between two groups. ^**^ *p* < 0.01, ^***^ *p* < 0.001

**FIGURE 3:**
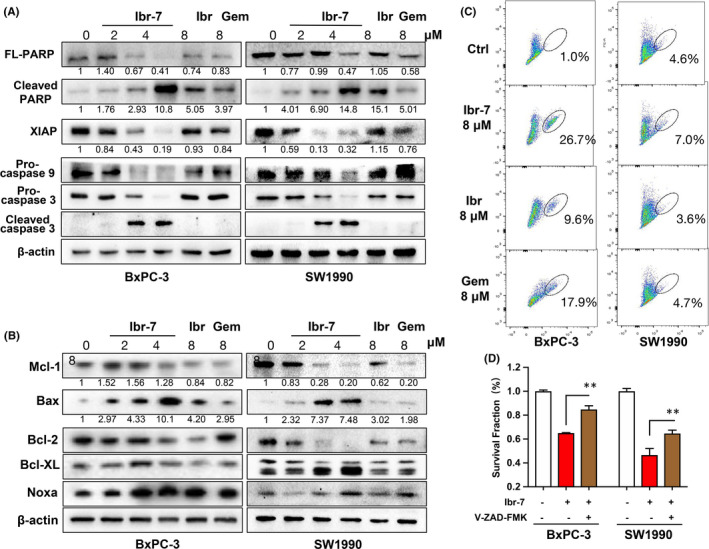
Mitochondria are involved in ibr‐7‐induced apoptosis in PDAC cells. (A) The effect of ibr‐7, ibrutinib and gemcitabine on apoptotic proteins. BxPC‐3 and SW1990 cells were treated with indicated concentrations for 48 h before Western blotting analysis. (B) The effect of ibr‐7, ibrutinib and gemcitabine on mitochondrial proteins. BxPC‐3 and SW1990 cells were treated with indicated concentrations of compounds for 24 h before Western blotting analysis. (C) Ibr‐7 disrupted mitochondrial membrane potential. BxPC‐3 and SW1990 cells were treated with indicated concentrations of compounds for 24 h before collection. Then, cells were stained by JC‐1 and analysed by flow cytometry. Three independent experiments were performed. (D) A pan‐caspase inhibitor V‐ZAD‐FMK was pretreated with both BxPC‐3 and SW1990 cells for 24 h, and cells were incubated with ibr‐7 for another 48 h before CCK‐8 assay. Three independent experiments were performed, and data were presented as mean ± SD. Student's t test was used to make a comparison between two groups. ^**^ *p* < 0.01

To validate the inhibitory effects of ibr‐7 on pancreatic cancer cells, Annexin V/PI was utilized to stain apoptotic cells. After 24 h treatment with ibr‐7 at a dose of 8 μM, the results of flow cytometric analysis showed that percentage of apoptotic cells reached to 73.7% 45.6% and 59.1% in BxPC‐3, SW1990 and CFPAC‐1 cells, respectively, comparing with 23.4%, 5.9% and 9.5% after exposure to ibrutinib at the same concentrations (Figure 2A). The occurrence of apoptosis was further validated by nuclear staining, showing apoptotic bodies after ibr‐7 treatment (Figure 2B). In addition, apoptotic proteins were examined after exposure to ibr‐7, ibrutinib or gemcitabine for 24 h. At the dose of 8 μM, ibrutinib and gemcitabine had only minor effects on XIAP, procaspase‐9 and cleaved caspase‐3. However, treatment with 8 μM of ibr‐7 could significantly downregulate the expression of full‐length PARP, XIAP, procaspase‐3 and 9 (Figure 3A), suggesting that ibr‐7 was more effective in the induction of apoptosis than either ibrutinib or gemcitabine in PDAC cells.

We then aimed to examine the integrity of mitochondrion after ibr‐7 treatment. The results showed that both ibr‐7 and ibrutinib markedly downregulated the expression of Mcl‐1, Bcl‐2 and Bcl‐xL. Noticeably, apparent elevated expression of Bax was only seen after ibr‐7 exposure but not ibrutinib, suggesting the activation of Bax in ibr‐7 treated BxPC‐3 and SW1990 cells (Figure 3B). Generally, activated Bax might demolish the function of mitochondrion; thus, we evaluated mitochondrial membrane potential using JC‐1 stain. After treatment with indicated compounds for 16 h, the proportion of red to green cells, which indicated depolarized membrane potential, significantly increased only after exposure to ibr‐7 (Figure 3C). These results suggested that mitochondria were involved in ibr‐7‐induced apoptosis. In addition, a pan‐caspase inhibitor V‐ZAD‐FMK was pretreated with BxPC‐3 and SW1990 cells before incubation with ibr‐7. As a result, an approximate 20% increase in survival cells was seen after V‐ZAD‐FMK pretreatment.

### 3.3 Ibr‐7 and gemcitabine synergistically inhibited the growth of PDAC cells through repressing mTOR/p79S6K pathway

**FIGURE 4:**
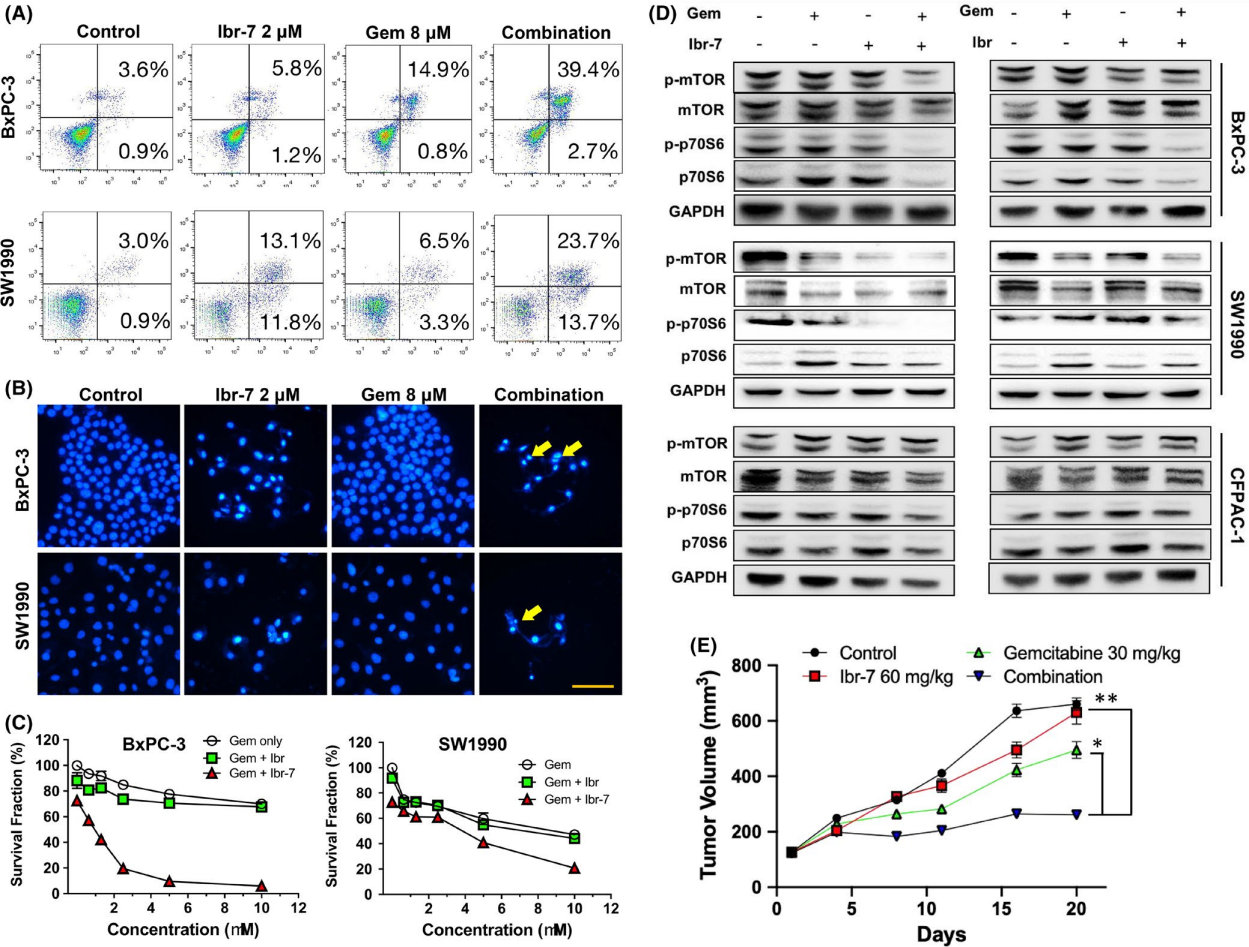
Ibr‐7 and gemcitabine had synergistic effects in killing PDAC cells. (A) Cells were treated with Ibr‐7, ibrutinib or the combination for 24 h before collection. Then, cells were stained by Annexin V/PI and analysed by flow cytometry. (B) Apoptotic bodies were observed after combinatorial treatment of ibr‐7 and gemcitabine. Cells were treated with 2 µM of ibr‐7, 8 µM of gemcitabine or the combination for 24 h before DAPI stain. Fluorescence was observed by using microscopy (Nikon Eclipse Ti). (C) The combinatorial effect of ibr‐7/ibrutinib and gemcitabine on BxPC‐3 and SW1990 cells in vitro. Cells were treated with the combination of various concentrations of gemcitabine and 2 µM of ibr‐7/ibrutinib for 48 h before CCK‐8 assay. (D) Combinatorial treatment with ibr‐7 and gemcitabine dramatically inhibit the activation of mTOR pathway. BxPC‐3, SW1990 and CFPAC‐1 cells were treated with indicated compounds for 8 h before Western blotting assay. (E) The combination of ibr‐7 and gemcitabine showed synergism in BxPC‐3 cells xenograft in nude mice. Gemcitabine, ibr‐7 or the combination were administrated intraperitoneally every two or three days. Tumour volumes were determined from calliper measurements of tumour length (L) and width (W) according to the formula (L × W2)/2. Three independent experiments were performed, and data were presented as mean ± SD. Student's t test was used to make a comparison between two groups. ^*^ *p* < 0.05, ^**^ *p* < 0.01

Gemcitabine has been used as the first‐line chemotherapy against pancreatic cancer in the last decades, whereas few agents showed synergistic effects when combined with gemcitabine in clinic trials. Therefore, it is in urgent need to explore novel agents that sensitize the efficacy of gemcitabine in PDAC cells. In our study, the combination treatment of ibr‐7 and gemcitabine resulted in 42.1% and 37.4% apoptotic cells in BxPC‐3 and SW1990 cells respectively (Figure 4A). In addition, the occurrence of apoptosis caused by the combination treatment of ibr‐7 and gemcitabine was validated by the appearance of apoptotic bodies (Figure 4B). At the same conditions, ibrutinib failed to sensitize gemcitabine in both BxPC‐3 and SW1990 cells in vitro, indicating the unique combinatorial effect of ibr‐7 and gemcitabine in PDAC cells (Figure 4C).

The combinatorial effect on mTOR/p70S6K pathway was also examined by treating cells with 2 μM of ibr‐7 and 8 μM of gemcitabine. The combination treatment rather than single treatment effectively downregulated the phosphorylation of mTOR and p‐70S6 in BxPC‐3, SW1990 and CFPAC‐1 cells (Figure 4D). In addition, the synergistic effect of ibr‐7 and gemcitabine was further recapitulated in the in vivo antitumour study. The combination treatment significantly repressed the growth of BxPC‐3 xenograft tumour, comparing with either ibr‐7 or gemcitabine alone (Figure 4E). Meanwhile, there was no significant difference in body weights between combination treatment and gemcitabine alone group, suggesting that toxicity caused by combination treatment was not enhanced by the addition of ibr‐7 (data not shown).

### 3.4 Ibr‐7 exerted its synergistic effects with gemcitabine via suppression of TRIM32

**FIGURE 5:**
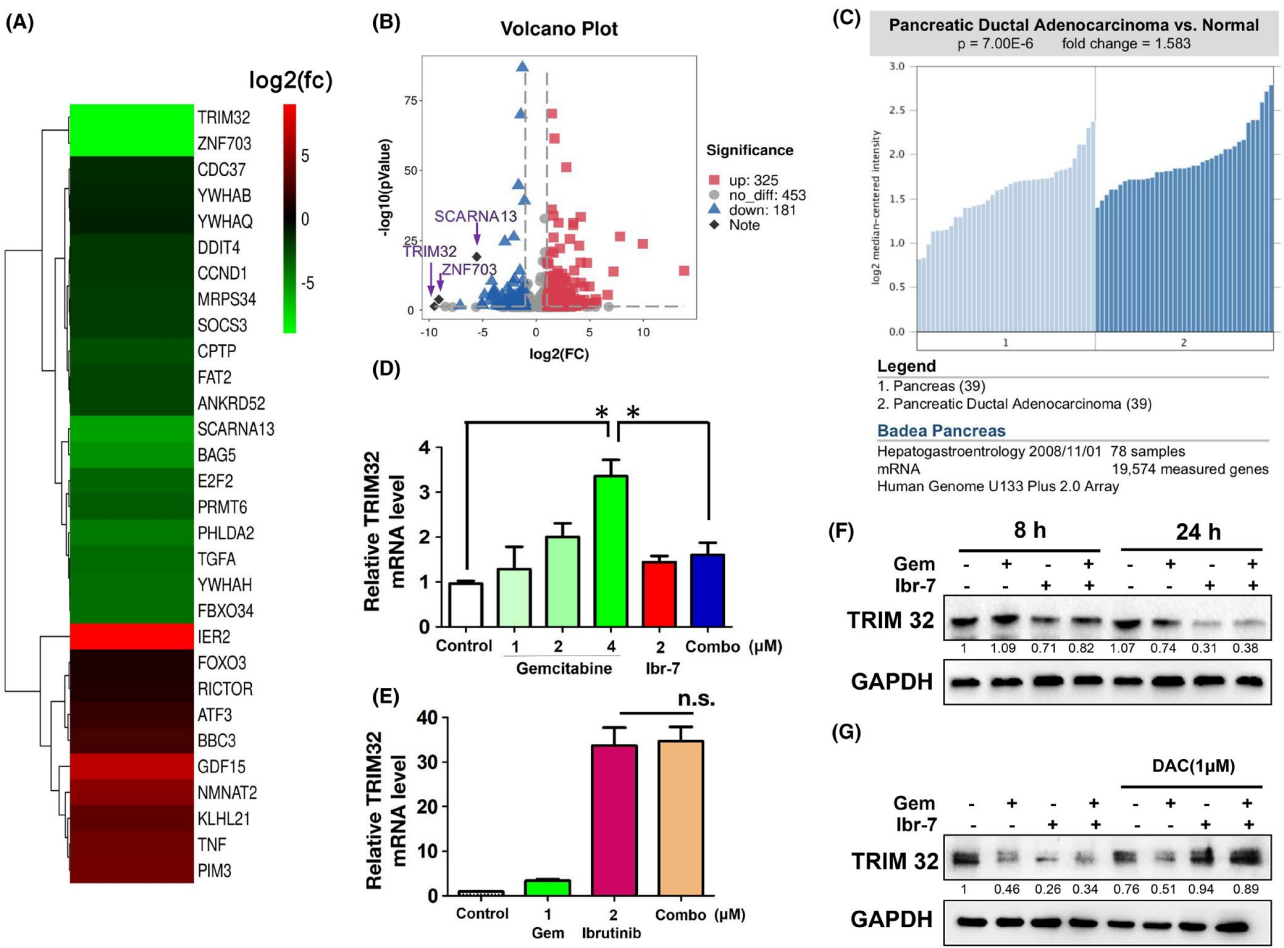
Differential expression of TRIM32 after combination treatment with ibr‐7 and gemcitabine. (A) Differential gene expression results from RNA‐sequencing data. (B) Volcano plot showing the effect of combinatorial treatment on differential gene expression (log2(fold change); ratio combination treatment group/gemcitabine group). The dotted dark line indicates the significance cut‐off (*p *<  0.05). Using a twofold change and the P and q values at 0.00 as a standard, 325 and 181 mRNAs were shown to be up‐regulated and downregulated in combinatorial treatment group. (C) TRIM32 was significantly higher in PDAC tissues than normal ones. TRIM32 mRNA level between PDAC patients and normal tissues were analysed using Cancer Genome Atlas (TCGA, https://www.cancer.gov) database. (D) BxPC‐3 cells were treated with gemcitabine, ibr‐7 or the combination for 8 h. Total RNA was extracted and underwent RT‐qPCR assay. (E) BxPC‐3 cells were treated with gemcitabine, ibrutinib or the combination for 8 h. Total RNA was extracted and underwent RT‐qPCR assay. (F) BxPC‐3 cells were treated with gemcitabine, ibr‐7 or the combination for 8 or 24 h before Western blotting assay. (G) Cells were pretreated with DAC for 2 h and then incubated with indicated compounds for 24. Protein expression of TRIM32 was observed by using Western blotting assay. Three independent experiments were performed, and data were presented as mean ± SD. Student's t test was used to make a comparison between two groups. n.s.=non‐significant, ^**^ *p* < 0.01

To find out key proteins that play decisive roles in the synergistic effect of ibr‐7 combined with gemcitabine, cluster of genes that differentially expressed in BxPC‐3 cells after gemcitabine or combination treatment were identified by using mRNA sequencing. A total of 506 mRNAs were shown to be significantly changed between combination and gemcitabine group. Using a twofold change and the P and q values at 0.00 as a standard, 325 and 181 mRNAs were shown to be up‐regulated and downregulated in combination treatment comparing with gemcitabine group respectively (Figure 5A–B). Notably, TRIM32 was the most significantly downregulated gene among these detected genes. Using Cancer Genome Atlas (TCGA, https://www.cancer.gov) database, TRIM32 mRNA was significantly increased in PDAC patients versus normal tissues (Figure 5C). In addition, the elevated expression of TRIM32 was closely associated with exposure to gemcitabine in a dose‐dependent manner, while either ibr‐7 or the combination treatment could restore the TRIM32 mRNA level (Figure 5D). On the contrary, ibrutinib could not suppress TRIM32, but dramatically increase the mRNA expression of TRIM32 (Figure 5E). Moreover, we aimed to determine the protein expression of TRIM32 after ibr‐7 or the combination treatment. As shown in Figure 5F, the protein level of TRIM32 considerably decreased after combination treatment for 24 h. To further understand the transcriptional regulation of TRIM32, cells were pretreated with decitabine (DAC), which was known as a DNA demethylating agent, for 2 h before incubation with ibr‐7, gemcitabine or the combination. As a result, pretreatment with dcecitabine allowed restoration of TRIM32 protein level after exposure to the combination treatment, suggesting that diminished TRIM32 mRNA transcription level was attributed to its DNA methylation (Figure 5G).

### 3.5 TRIM32 played a key role in the progression of PDAC cells

**FIGURE 6:**
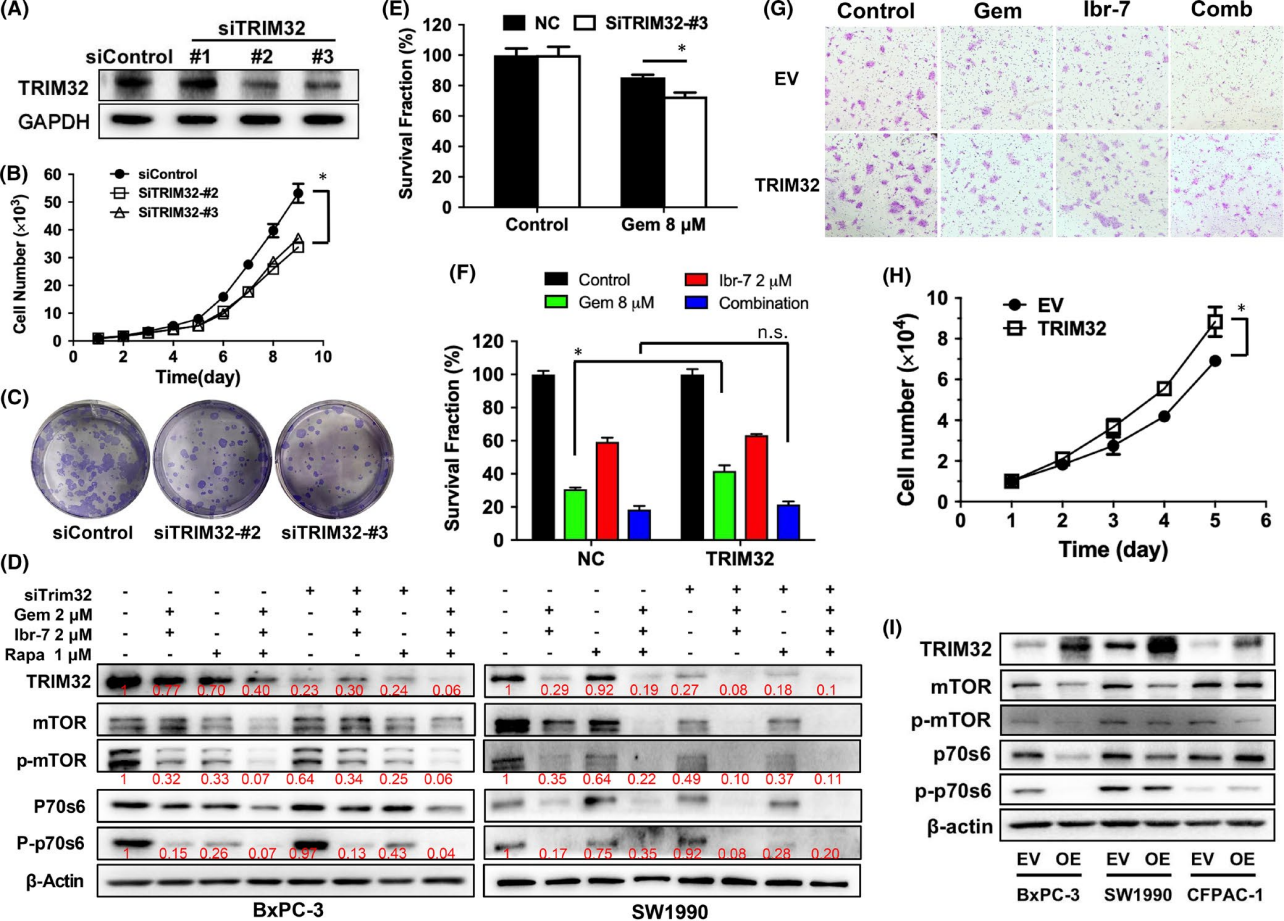
Role of TRIM32 in malignant properties of PDAC cells. (A) BxPC‐3 cells were transfected with small interfering RNA (siRNA) targeting TRIM32 (SiTRIM32) or scramble siRNA (SiControl). After cells were transfected for 24 h, proteins were collected and analysed by Western blotting assay. (B) BxPC‐3 cells were seeded in 96‐well plates at a density of 1 × 10^3^/well and cultured for 24 h. After transfection with siTRIM32 or siControl, BxPC‐3 cell number was counted every day for 9 days. (C) BxPC‐3 cells were seeded in 6‐well plates at a density of 1 × 10^3^/well and cultured for 24 h. After transfection with siTRIM32 or siControl, the supernatant was removed and cells were cultured for another two weeks. Then, cells were fixed with 4% paraformaldehyde for 15 min and stained with Giemsa solution for 15 min at room temperature. Visible colonies were imaged with a ChemiDoc XPS system. (D) BxPC‐3 and SW1990 cells were treated with siTRIM32 or indicated agents for 8 h before Western blotting assay. Gem =gemcitabine, Rapa =rapamycin. (E) BxPC‐3 cells were seeded in 96‐well plates at a density of 6 × 10^3^/well and cultured for 24 h. Then, cells were transfected with siTRIM32 or siControl for another 24 h. Cells were treated with 8 μM of gemcitabine for 24 h before CCK‐8 assay. (F) BxPC‐3 cells were seeded in 96‐well plates at a density of 6 × 10^3^/well and cultured for 24 h. After transfection with TRIM32, cells were treated with ibr‐7 (2 µM), gemcitabine (8 µM, Gem) or the combination for 24 h before CCK‐8 assay. (G) BxPC‐3 cells were transfected with TRIM32 and incubated with a combination of ibr‐7 (2 µM), gemcitabine (8 µM, Gem) or the combination for 24 h before migration assay. (H) BxPC‐3 cells were cultured in 24‐well plates at a density of 1 × 10^4^/well and transfected with TRIM32 for 24 h. Cells were incubated in culturing medium for 5 days, and cell number was counted every day by using Counterstar (Shanghai, China). (I) BxPC‐3, SW1990 and CFPAC‐1 cells were transfected with empty vector (EV) or TRIM32 (OE, overexpression) and cultured for 48 h. Then, cells were lysed, and indicated proteins were examined by Western blotting assay. Three independent experiments were performed, and data were presented as mean ± SD. Student's t test was used to make a comparison between two groups. ^*^ *p* < 0.05. n.s.=non‐significant difference

To elaborate the role of TRIM32 in malignant characteristics of PDAC, we used RNA interference to diminish the expression of TRIM32 (Figure 6A). As a result, knocking down of TRIM32 not only impeded the proliferation of BxPC‐3 cells, but also suppressed the colony formation of BxPC‐3 cells (Figure 6B–C). Next, we intended to understand the influence of TRIM32 silencing on mTOR/p70S6K pathway. In BxPC‐3 cells, silencing TRIM32 significantly suppressed the phosphorylated p‐p70S6, without affecting total or phosphorylated mTOR. In SW1990 cells, the inhibitory effects on mTOR were also prominent after TRIM32 silencing (Figure 6D). Moreover, either TRIM32 silencing or the addition of rapamycin could potentiate the inhibition of mTOR/p70S6K pathway after the combination treatment with ibr‐7 and gemcitabine. These data suggested a positive feedback loop between TRIM32 and mTOR/p70S6K pathway.

Then, we aimed to investigate the role of TRIM32 in drug sensitivity of PDAC cells. Interestingly, silencing of TRIM32 could enhance the cytotoxicity of gemcitabine in BxPC‐3 cells (Figure 6E), while overexpression of TRIM32 partially attenuated the inhibitory effects of gemcitabine single treatment but not the combination treatment (Figure 6F). In addition, excessive TRIM32 could enhance the invasiveness of BxPC‐3 cells (Figure 6G) and meanwhile accelerated the proliferation of BxPC‐3 cells (Figure 6H). Interestingly, in three PDAC cells with established sustained overexpression of TRIM32, total or phophorylated mTOR was found to be downregulated compared with those transfected with empty vector (Figure 6I).

## 4 DISCUSSION

In the era of precision medicine, targeted therapy for pancreatic cancer is facing critical challenges. On one hand, the stroma of PDAC microenvironment provides a natural barrier against most of therapeutic drugs and creates metastatic niche for tumour progression.^21^, ^22^ On the other hand, PDAC cells are naturally insensitive to current chemotherapy or targeted drugs.^23^, ^24^ Ibr‐7 was a derivative of ibrutinib that obtained in our previous work.^17^ Preliminary studies showed that ibr‐7 could inhibit the proliferation of PANC‐1 and Capan2 cells in vitro, and enhanced radiosensitivity in a p‐EGFR dependent manner.^18^ However, several questions remained to be fully illustrated: the underlying mechanisms of ibr‐7’s inhibitory activity in PDAC cells; whether ibr‐7 was capable to improve the efficacy of gemcitabine; and the potential molecular targets that sensitizing gemcitabine in PDAC cells. Herein, we not only explored the molecular mechanisms of anti‐PDAC activity of ibr‐7 but also investigated the regulatory loop between TRIM32 and mTOR/p70S6K pathway, thus preliminarily demonstrating the potential role of TRIM32 as a novel molecular target for sensitizing gemcitabine in PDAC cells.

First of all, we examined the anti‐proliferation activity of ibrutinib and ibr‐7 in four PDAC cell lines in vitro. The IC50 values of ibr‐7 ranged from 0.8 to 3.5 μM, which were 10‐fold to 60‐fold less than that of ibrutinib (Table 1). To explore the underlying mechanisms, we examined key proteins that are closely correlated with cell survival. mTOR and ERK are two downstream pathways of KRAS, which are essential for PDAC cell proliferation, and the inhibition of either pathway might induce the hyperactivation of the other one.^25^, ^26^ In our study, ibr‐7 substantially abrogated the activation of both mTOR and ERK signalling pathway, resulting in its superior anti‐proliferation activity than ibrutinib. The activation of mTOR pathway might influence mitochondrial proteins and protect cells from mitochondrial‐dependent apoptosis.^27^, ^28^ For instance, AKT activation could upregulate Mcl‐1, leading to sequestered BIM and inactivation of Bax.^29^ It was also reported that mTOR inhibitors could induce the suppression of Mcl‐1.^30^, ^31^ In the present study, the expression of Mcl‐1 was closely related to p‐mTOR after exposure to indicated compounds in SW1990 but not BxPC‐3 cells, suggesting different regulation patterns in two tested cell lines. On the contrary, the expression of Bax was unanimously activated after ibr‐7 treatment in both BxPC‐3 and SW1990 cell lines, indicating the participation of mitochondria in ibr‐7‐induced apoptosis.

Since gemcitabine is the standard chemotherapy treatment for pancreatic cancer, we speculated that whether ibr‐7 could potentiate the antitumour efficacy of gemcitabine. As a result, ibr‐7, but not ibrutinib, exerted strongly synergism against PDAC cells when combined with gemcitabine both in vitro and in vivo (Figure 4C–E). By analysing the differential gene expression, TRIM32 was the most downregulated gene in 959 differential genes while comparing combinatorial treatment and gemcitabine single treatment (Figure 5A–B). Indeed, TRIM32 was significantly downregulated after the combinatorial treatment of gemcitabine and ibr‐7, but not ibrutinib (Figure 5D–E). It was reported that TRIM32 played a pro‐apoptotic role via destabilizing mitochondrial membrane potential and degradation of XIAP in normal cells.^32^, ^33^ In the context of cancer, TRIM32 was able to promote the proliferation and motility of lung, gastric, squamous cancer cells or contribute to cisplatin resistance in colorectal cancer, whereas the role of TRIM32 in PDAC cells remained unknown.^34^, ^35^, ^36^, ^37^, ^38^ In our study, we found that the combination treatment of ibr‐7 and gemcitabine could diminish TRIM32 at a transcriptional level, probably through enhancing the methylation status of TRIM32 (Figure 5F,G).^39^, ^40^

Based on the aforementioned data, we assumed that the direct inhibition of mTOR/p70S6K pathway caused by ibr‐7 probably contributed to the suppression of TRIM32. In another aspects, whether TRIM32 possessed a reciprocal feedback loop with mTOR/p70S6K in PDAC cells remained to be illuminated. It was reported that TRIM32 reduced PI3K‐Akt‐FoxO signalling by promoting plakoglobin‐PI3K dissociation in muscle atrophy,^41^ and TRIM32 deficiency was found to cause hypoactive mTOR in autism spectrum disorder mice model.^42^ In gastric cancer cells, TRIM32 was able to promote the AKT activity and glucose transportation.^43^ In this work, knocking down TRIM32 significantly impeded the proliferation and diminished the colony formation of BxPC‐3 cells. Moreover, silencing TRIM32 could significantly suppressed the phosphorylated p‐p70S6 or mTOR in BxPC‐3 and SW1990 cells, respectively (Figure 6D), which was in consistent with previous reports. Nevertheless, different situations were found in stable transfected PDAC cells with overexpression of TRIM32. Although overexpression of TRIM32 enhanced the invasion and growth of BxPC‐3 cells, a repression of either mTOR or p70S6 was seen in all three PDAC cell lines (Figure 6I). This phenomenon could be explained by the functional role of TRIM32 as an E3 ligase, the residual of which resulted in increased proteasomal degradation in PDAC cells.^44^, ^45^

In summary, our study showed that ibr‐7 alone or combined with gemcitabine exhibited potent anti‐PDAC activity both in vitro and in vivo via the suppression of mTOR/p70S6K pathway, accompanied with significant downregulation of TRIM32. TRIM32 could form a positive feedback loop with mTOR/p70S6K, dictate malignant properties of PDAC cells and finally affected the drug sensitivity of gemcitabine. Therefore, our study not only investigated the underlying mechanisms of ibr‐7 in PDAC cells but also shed light on the potential role of TRIM32 as a molecular target to sensitize the efficacy of gemcitabine in treating PDAC.

## CONFLICT OF INTEREST

The authors confirm that there are no conflicts of interest.

## AUTHOR CONTRIBUTION

**Bo Zhang:** Conceptualization (equal); Writing – original draft (lead); Writing – review & editing (lead). **You‐you Yan:** Data curation (equal); Formal analysis (equal); Investigation (equal); Methodology (equal). **Yangqin Gu:** Investigation (equal); Methodology (equal); Validation (equal). **Fei Teng:** Methodology (equal); Validation (equal). **Xu Lin:** Software (equal). **Xinglu Zhou:** Resources (equal). **Jinxin Che:** Visualization (equal). **Xiaowu Dong:** Investigation (equal); Writing – original draft (equal). **Lixin Zhou:** Conceptualization (equal); Resources (equal). **Nengming Lin:** Conceptualization (lead); Resources (lead).
